# Treatment of Cutaneous Leishmaniasis Caused by *Leishmania aethiopica*: A Systematic Review

**DOI:** 10.1371/journal.pntd.0004495

**Published:** 2016-03-03

**Authors:** Johan van Griensven, Endalamaw Gadisa, Abraham Aseffa, Asrat Hailu, Abate Mulugeta Beshah, Ermias Diro

**Affiliations:** 1 Department of Clinical Sciences, Institute of Tropical Medicine, Antwerp, Belgium; 2 Armauer Hansen Research Institute (AHRI), Addis Ababa, Ethiopia; 3 School of Medicine, Addis Ababa University, Addis Ababa, Ethiopia; 4 World Health Organization, Addis Ababa, Ethiopia; 5 Department of Internal Medicine, University of Gondar, Gondar, Ethiopia; Institut Pasteur de Tunis, TUNISIA

## Abstract

*Leishmania aethiopica* is the etiological agent of cutaneous leishmaniasis (CL) in Ethiopia and can cause severe and complicated cases such as diffuse CL (DCL), mucocutaneous leishmaniasis or extensive CL, requiring systemic treatment. Despite the substantial burden, evidence-based treatment guidelines are lacking. We conducted a systematic review of clinical studies reporting on treatment outcomes of CL due to *L aethiopica* in order to help identify potentially efficacious medications on CL that can be taken forward for clinical trials. We identified a total of 24 records reporting on 506 treatment episodes of CL presumably due to *L aethiopica*. The most commonly used drugs were antimonials (n = 201), pentamidine (n = 150) and cryotherapy (n = 103). There were 20 case reports/series, with an overall poor study quality. We only identified two small and/or poor quality randomized controlled trials conducted a long time ago. There were two prospective non-randomized studies reporting on cryotherapy, antimonials and pentamidine. With cryotherapy, cure rates were 60–80%, and 69–85% with antimonials. Pentamidine appeared effective against complicated CL, also in cases non-responsive to antimonials. However, all studies suffered from methodological limitations. Data on miltefosine, paromomycin and liposomal amphotericin B are extremely scarce. Only a few studies are available on DCL. The only potentially effective treatment options for DCL seem to be antimonials with paromomycin in combination or pentamidine, but none have been properly evaluated. In conclusion, the evidence-base for treatment of complicated CL due to *L aethiopica* is extremely limited. While antimonials remain the most available CL treatment in Ethiopia, their efficacy and safety in CL should be better defined. Most importantly, alternative first line treatments (such as miltefosine or paromomycin) should be explored. High quality trials on CL due to *L aethiopica* are urgently needed, exploring group sequential methods to evaluate several options in parallel.

## Author Summary

Cutaneous leishmaniasis (CL) refers to skin ulcers caused by the *Leishmania* parasite, which is transmitted by the bite of sandflies. In Ethiopia, CL is caused by the *Leishmania aethiopica* parasite. CL in Ethiopia can be associated with severe and complicated disease such as diffuse CL (DCL), which presents with multiple skin lesions spread over the body. For such severe cases, topically applied treatment is not sufficient and systemic treatment (given in tablets or via injections) is required. Although the total number of patients that suffer from CL in Ethiopia is high, there are no evidence-based treatment guidelines. We conducted a systematic review of clinical studies reporting on treatment outcomes of CL due to *L aethiopica*. We identified a total of 24 records reporting on 506 treatment episodes of CL presumably due to *L aethiopica*. The most commonly used drugs were antimonials, pentamidine, and cryotherapy. Most studies were case reports or case series. There were two small clinical trials and two prospective non-randomized studies. However, all studies had some methodological limitations. With cryotherapy, cure rates were 60–80%, and 69–85% with antimonials. Pentamidine appeared effective against complicated CL, also in cases that did not improve on antimonials. Data on miltefosine, paromomycin and liposomal amphotericin B are extremely scarce. Only a few studies were available on DCL. The evidence-base for treatment of complicated CL due to *L aethiopica* is extremely limited. While antimonials remain the most available CL treatment in Ethiopia, more data on the efficacy and safety in CL are needed. High quality trials on CL due to *L aethiopica* are urgently needed, evaluating treatments beyond antimonials. Interesting options in the short term are drugs such as miltefosine, paromomycin and liposomal amphotericin B, as they are currently available in Ethiopia for treatment of visceral leishmaniasis.

## Introduction

Cutaneous leishmaniasis (CL) is a chronic infectious skin disease caused by a group of protozoan parasites of the *Leishmania* genus. The parasites are transmitted to humans via the bite of phlebotomine sandflies and predominantly target reticulo-endothelial cells [1]. CL can present with a spectrum of clinical manifestations. Ulcerative skin lesions occurring at the site of the bite of the sandfly is the most common cutaneous manifestation (localized CL—LCL). While usually healing spontaneously after several months, it remains disfiguring and stigmatizing and often heals with scarring. There are several more rare forms like diffuse CL (DCL), which is often difficult to treat [1]. Mucosal leishmaniasis (ML) or mucocutaneous CL (MCL) refers to an often destructive form with mucosal inflammation, which has been mainly reported in the New World—in association with *L braziliensis* [1,2]—but also in the Old World [3]. At the global level, around one million cases of CL occur annually [4]. In the New World, *L braziliensis* causes the largest CL burden, with Brazil most severely affected. In the Old World, most cases are found in the Middle East, North Africa, the Indian subcontinent and Central Asia [4].

While CL in the Old world is predominantly caused by *L tropica* and *L major*, it is still estimated that several ten thousands of cases are due to *L aethiopica*. These predominantly occur in Ethiopia, and more exceptionally in Kenya. Within Ethiopia, the annual CL burden is estimated at around 20.000 to 40.000 cases per year [4], of which 99% is thought to be due to *L aethiopica* [5]. A recent study estimated almost 30 million of Ethiopians to be at risk for CL [6]. CL in Ethiopia is a zoonotic disease, mainly occurring in the highland regions, involving rock hyraxes as reservoir. The disease predominantly affects children, adolescents and young adults [7–10]. In the Northern part of Ethiopia, HIV coinfection rates of 5.6% have been reported [7]. An outbreak of CL has recently been described [11].

Clinical manifestations of *L aethiopica* are particularly diverse and pleotropic, and a high genetic diversity has been documented as well [12]. Localized CL (LCL) is the most frequent manifestation, while mucocutaneous (MCL) and diffuse CL (DCL) is relatively common [7]. Compared to LCL, MCL is reportedly less responsive to treatment and is more disfiguring [1]. DCL is notorious for its chronic and progressive course and non-responsiveness to the common antileishmanial drugs. It is characterized by highly parasitized nodular lesions spread throughout the body and the failure to mount an effective antileishmanial immune response. Even if lesion regression can be obtained with chemotherapy, most cases of DCL will relapse after treatment discontinuation [1]. Outside Ethiopia, DCL is rare and occasionally seen linked to *L amazonensis* and *L mexicana* [1,13].

Since its occurrence is restricted to almost a single country with limited resources, research on this species has been relatively limited, especially over the last 10 to 20 years when commitments to combat neglected tropical diseases have been enhanced and major scientific and technological breakthroughs have occurred. In terms of treatment of *L aethiopica*, the evidence base remains extremely limited. A Cochrane review published in 2008 identified not a single randomized clinical trial dedicated to CL in Ethiopia [14]. Nevertheless, *L aethiopica* has particular features (the frequent occurrence of DCL and MCL) that imply a potentially higher need of systemic therapy, as compared to other species such as *L major or L tropica*. It is clear that randomized clinical trials are highly needed and should be undertaken. It is however less clear which interventions should be selected for prioritization in these studies. We conducted a systematic review including any type of clinical study reporting on outcomes in humans of drug treatment of CL due to *L aethiopica*, in order to help identify potentially efficacious medications for CL that can be taken forward in clinical trials. Laboratory studies evaluating drug susceptibility of *L aethiopica* against currently available antileishmanial drugs were reviewed as well.

## Methods

### Types of studies and search strategy

This review was conducted in line with the PRISMA guidelines; the PRISMA checklist was completed (See S1 Checklist) [15]. The sources searched and the search terms used are presented in Tables 1 and 2. Additional publications were identified by reviewing the reference lists of selected papers and by contacting experts in the field. As a first step, titles and abstracts were reviewed independently by two reviewers (JvG and ED) and those selected by at least one reviewer were included for evaluation of the full text. The final selection of studies to be included for data extraction was done independently by two reviewers (JvG and ED), with discrepancies solved by consensus. The scope of the review was any study in humans reporting on treatment outcomes of CL due to confirmed or presumed *L aethiopica*. There was no selection based on patient age, sample size, study design, language or period. There was no selection by type of intervention (systemic, local, physical or other). Since the aim of this review was to prioritize interventions to be taken forward in phase III clinical trials, early studies on traditional therapies were not considered. No specific criteria were set for the outcome (treatment response), besides that the paper had to include at least some information on evolution after treatment. On some occasions, authors were contacted for additional information or clarifications. No specific protocol was developed for this systematic review.

**Table 1:** Search terms and date of first and latest search for the different electronic databases used.

Source	Search terms	Date of first and latest search^a^
Pubmed/Medline	See Table 2	2013/11/12–2015/09/20
Cochrane Register of Studies Cochrane Central Register of Controlled Trials (CENTRAL) CENTRAL		2015/09/20
Clinical trial.gov	Cutaneous leishmaniasis or cutaneous leishmania	2013/11/12–2015/09/20
Google scholar	(cutaneous leishmaniasis) (ethiopia OR aethiopica OR ethiopica) (treatment OR therapy)	2013/11/12–2015/09/20

^a^ First date is the date the search started; last date is the date the search was updated

**Table 2:** Medline search strategy.

Nr	Search terms
**#1.**	Leishmaniasis, Cutaneous/complications [Mesh]
**#2.**	Leishmaniasis, Cutaneous/drug therapy [Mesh]
**#3.**	Leishmaniasis, Cutaneous/epidemiology [Mesh]
**#4.**	Leishmaniasis, Cutaneous/therapy [Mesh]
**#5.**	Leishmaniasis, Cutaneous/surgery [Mesh]
**#6.**	Leishmaniasis/prevention and control [Mesh]
**#7.**	1-6/or
**#8.**	Ethiopia [Mesh]
**#9.**	Ethiopica (ALL) OR aethiopica (ALL)
**#10.**	8 or 9
**#11.**	7 and 9
**#12.**	therapy
**#13.**	treatment
**#14.**	Management
**#15.**	therapeutic use
**#16.**	drug therapy
**#17.**	drug treatment
**#18.**	clinical care
**#19.**	cryotherapy
**#20.**	Cryosurgery
**#21.**	12-20/or
**#22.**	leishmaniasis or leishmania or (oriental sore)
**#23.**	(visceral leishmaniasis) OR Leishmaniasis, visceral [MESH])
**#24.**	22 and 10
**#25.**	24 not 23
**#26.**	21 and 25
**#27.**	11 or 26

### Data extraction and analysis

Data were extracted into pre-piloted tables by one reviewer (JvG) and verified by a second reviewer (ED), with discrepancies solved by consensus. Key outcome data were verified by duplicate extraction. The following information was extracted: 1) patient characteristics: age, sex, duration of lesions, type of CL (LCL, MCL or DCL); 2) whether the diagnosis was parasitologically confirmed and whether species identification was done; 3) travel-related or not; 4) treatment details; 5) treatment response and definitions used; 6) adverse events associated with the intervention; 7) relevant information relating to study quality or interpretation. Based on previously conducted SR on New World CL [14], we anticipated that no or very few (high quality) clinical trials would be found, but rather expected a range of small, generally non-randomized (low quality) studies, all with obvious study limitations. We used the Newcastle-Ottawa Scale (NOS) to assess the quality of nonrandomized studies [16]. Three broad perspectives were assessed: the selection of the study groups; the comparability of the groups; and the ascertainment of the outcome of interest. Clinical trials were evaluated as per standard quality assessment tool for randomized controlled trials in line with recommendations in the Cochrane handbook [17]. Given the nature of extracted data, only simple descriptive analysis was conducted, summarizing the individual studies.

## Results

### Characteristics of the selected manuscripts and strengths and weaknesses

**Fig 1:**
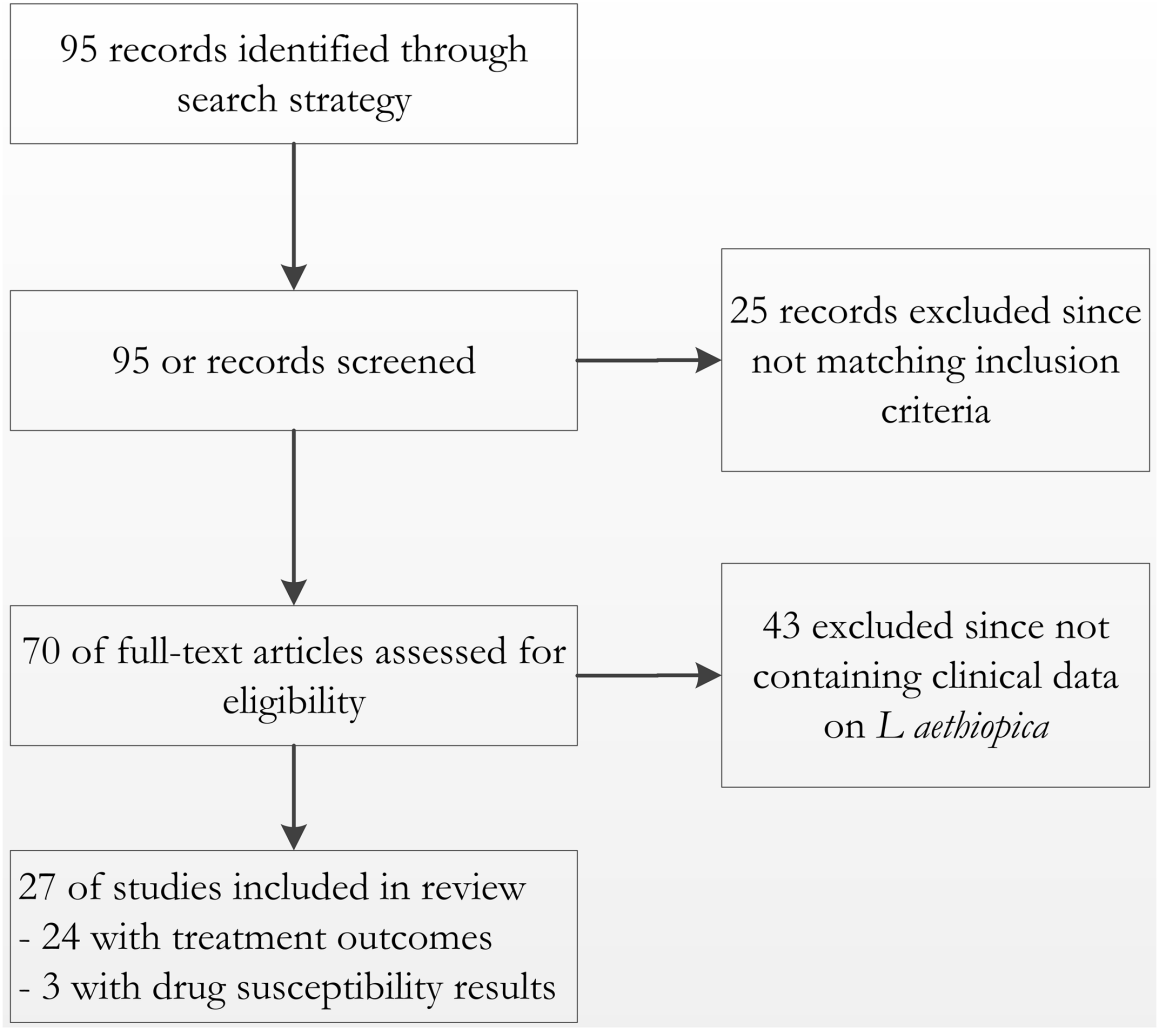
Overview of records identified, screened, reviewed and included in the review.

Out of a total of 95 studies or reports identified and screened, only 27 provided information relevant to our topic (Fig 1). This comprised 13 case series and seven case reports, generally clinical information collected as part of clinical practice. Most of these were reported over 30 to 40 years ago. There were two more recent prospective observational studies whereby a study protocol had been written and patient consent was requested. Since treatment allocation was based on CL type and/or severity, no true comparison in efficacy could be made. Only two small clinical trials were identified, one on tuberculosis drugs and pentamidine reported in 1981 and a small placebo-controlled trial on itroconazole reported in 1990. Three additional studies yielded relevant drug susceptibility information.

In total, outcomes of 506 treatment episodes were reported (excluding one report because of potential overlap). Most commonly used drugs were antimonials (n = 201), pentamidine (n = 150) and cryotherapy (n = 103). The majority of studies reported on patients being treated in *L aethiopica* endemic regions (one in Kenya, the other in Ethiopia) but only three of these studies did species identification in all reported cases. There were three cases of migrants treated in Europe or Israel. We identified nine studies on LCL cases, seven on DCL and two on MCL cases. In addition, three included LCL and DCL cases, two included LCL and MCL and DCL cases and one included LCL, MCL and DCL patients.

Most studies had obvious limitations, beyond the descriptive nature. Definitions of treatment outcomes varied across studies and were often not clearly defined. Sample size was often small, follow-up short and information on patient characteristics often limited. In formal quality assessment, the non-randomized studies performed poorly, with a median score of three (maximum score is eight), see Table 3. Only the two prospective studies reached a score of five. The oldest clinical trial on tuberculosis drugs and pentamidine had a high risk for blinding of participants/staff and outcome assessment, an unclear risk for bias for random sequence generation, allocation concealment and selective reporting and a low risk for incomplete outcome data. The trial on itraconazole had a low risk for random sequence generation, allocation concealment and blinding of participants/staff and outcome assessment, an unclear risk for bias for selective reporting and a high risk of bias for incomplete outcome data.

**Table 3:** Assessment of study quality of non-randomized studies using the Newcastle-Ottawa Scale (NOS).

	Treatment group representative	Control group representative	Treatment details given	Outcome not pre-existing	Comparability of groups	Independent outcome ascertainment	Follow up time (≥ 3 months)	Loss to follow up (<10%)	Total score
[7]	*	*	*	*	0	0	*	0	5
[46]	*	*	*	*	0	0	*	0	5
[5]	0	0	*	*	0	0	0	0	2
[47]	0	0	*	*	0	0	*	*	4
[48]	0	0	0	*	0	0	*	*	3
[49]	0	0	0	*	0	0	0	*	2
[50]	0	0	*	*	0	0	0	0	2
[51]	0	0	0	*	0	0	0	0	1
[52]	0	0	*	*	0	0	0	0	2
[53]	0	0	*	*	0	0	0	0	2
[54]	0	0	*	*	0	0	0	0	2
[55]	0	0	*	*	0	0	*	*	4
[56]	0	0	*	*	0	0	0	*	3
[57]	0	0	0	*	0	0	0	0	1
[58]	0	0	*	*	0	0	*	*	4
[59]	0	0	*	*	0	0	0	*	3
[60]	0	0	*	*	0	0	0	0	2
[61,62]	0	0	*	*	0	0	0	*	3
[21]	0	0	*	*	0	0	*	*	4
[63]	0	0	*	*	0	0	0	*	3
[53]	0	0	*	*	0	0	0	*	3
[64]	0	0	0	*	0	0	0	0	1

Three broad perspectives were assessed: the selection of the study groups; the comparability of the groups; and the ascertainment of the outcome of interest. Stars (*) indicate higher quality.

### Clinical studies on LCL and MCL treatment

We identified a total of 17 clinical studies reporting on treatment of CL or MCL (presumably) due to *L aethiopica*, relating to 384 treatment episodes (Table 4). There were two small clinical trials, two prospective non-randomized studies and 13 case reports/series. All but two included less than 100 patients. Species identification was systematically done in only six reports. One small placebo-controlled randomized clinical trial (n = 14) evaluated oral itraconazole for LCL and DCL and found it as effective as placebo. In another clinical trial, isoniazid, rifampicin and amithiozone were compared with pentamidine injections. While only one out of six patients improved with the former treatment, all six cases on pentamidine demonstrated improvement. However, no species identification was done and half of the cases were not parasitologically confirmed.

**Table 4:** Clinical studies on cutaneous and mucocutaneous leishmaniasis due to *L. aethiopica*.

Ref	Study design; country; year; study population^a^	Patient characteristics	Drug/Intervention	N	Efficacy	Comments
**Clinical trials**						
[65]	Clinical trial, ALERT	Each group: 1 single LCL	INH 300 mg, rifampicin	6	Clinically improved: 1 (at	Case that improved
	Ethiopia; 1981	histologically diagnosed	600 mg and amithiozone		8 weeks—EOT)	was smear(-) at start
	No species identification	smear(-)/LST(+); 3 multiple	150 mg for at least 8 weeks			(histologically
		LCL smear(+)/ LST(+);	Pentamidine	6	All 6 clinically improved	diagnosed)
		2 multiple LCL smear	dimethansulphonate 4		and skin smear negative	Treatment allocation
		pos(+)/LST(-)	mg/kg 15 doses alternative		EOT (4 weeks)	not clear: random?
		No further details	days IM			
[21]	Placebo-controlled	Age 12–48 years	Itraconazole 4 x 50 mg PO	7	EOT: active lesion 7;	Itraconazole generally
	randomized double		daily for 4 weeks		clinically improved 0	well tolerated
	blind clinical trial				M1 FU: 2/5 improved	None of the DCL
	ALERT/AHRI				Parasite culture(-): 2/5	patients improved
	Ethiopia; 1990		Placebo	7	EOT: active lesion 5;	Generation of
	CL (10) and DCL (4)				clinically improved 2	randomization and
	No species identification				M1 FU: 4/7 improved	concealment of
					Parasite culture(-): 4/7	allocation not
						clear
**Main focus antimonials or cryotherapy**						
[7]	Prospective evaluation	75.3% males;	LCL: SSG local injection	154	6M overall outcome:	13 of 167 not treated
	in free NGO CL clinic;	Age 5–14: 40 (24%)	every 3 days/4 weeks		69% (106) cure; 15%	(11 mild; 2 severe CL)
	Tigray region, Ethiopia	Age 15–44: 113 (68%)	DCL, MCL, RCL, PKDL,		(23) failure; 14% (21)	Cure: free from clinical
	1/2005-7/2007		relapse: MA 20 mg/kg		relapse; 4 stopped	and microbiological
	No species identification		for 30 days IV		systemic SSG for	All cases presumably
					toxicity	parasitologically
	LCL (123; 13 not treated)			110	0 non-response, 9 relapse	confirmed (?)
	DCL (11)			11	8 non-response; 3 relapse	HIV coinfection 5.6%
	MCL (29)			29	13 non-response; 7 relapse	(5/89): all relapsed
	ML (1)			1	1 non-response	11 of 21 relapses:
	PKDL (1)			1	1 non-response	no response to
	RCL (2)			2	0 non-response, 2 relapse	prolonged IV SSG
	DCL; SSG resistant		Pentamidine isothionate	8	7/8 (87% cured) by M6	No intolerance to
	relapse (1)		(if non response to SSG)		Cure in MCL and MCL	pentamidine
	MCL; SSG resistant		4 mg/kg every		DCL case initially	
	relapse (6)		second day till negative		clinically improved;	
	RCL; SSG resistant relapse		aspirate + clinical resolution		followed by relapse	
	relapse (1)		(~ 20 days)			
[46]	Non-randomized prospective	Mean age 18.4 years	Liquid nitrogen weekly	103	Cure: 83 (80.6%)	Per protocol cure rate
	prospective study	Male 53/103	until cure (3–4 sessions		Non-response: 6 (5.8%)	Cryotherapy: 93.3%
	South-Ethiopia (Silti);	Mean lesion duration:	10–30 sec per lesion		Dropout: 14 (13.6%)	SSG: 89.5%
	11/2008-6/2009	8.5 months	per visit)			Cure (M3): ulcers:
	LCL and MCL cases (n = 14)	Mean age 19.6 years	SSG 20 mg/kg/day for 30	20	Cure: 17 (85.0%)	complete scarring
	(active & passive case	Male 14/20	days IM—FU?		Non-response: 2 (10.0%)	Nodules: flattening
	finding)	Mean lesion duration:	(SSG if lesions: > 3; or		Dropout: 1 (5.0%)	no inflammation
		13.1 months	esthetically sensitive			
			areas; or MCL)			
[5]	Case series presented	Total 559 cases	Liquid nitrogen	559	340 discharged cured	Partly overlapping with
	at consultative meeting	341 male, 218 female	Duration variable		114 still on treatment	record above [46]
	ALERT/AHRI	Age range 2–70 years	4–12 weeks up to >		105 interrupted	
	Addis Abeba, 2001–2006	79% lesion in face	60 weeks		(16 failing cases found	
	Confirmed CL cases	79% LCL, 4% multiple			with other pathology)	
	Unclear about	CL; 17% MCL				
	species identification					
[47]	Case series, Kenya—LCL	Male adults	SSG 18–20 mg/kg IV twice	3	All patients improved and	No severe toxicity
	1981–1982	Age: 18, 21, 22 years	daily 30 days		parasite negative during	(mainly thrombosis and
	Cases unresponsive	Lesion duration 5, 7,			treatment	transient ECG and liver
	to SSG	9 months			All clinically cured (by	test abnormalities)
	Species identification in				M2-M6; one relapse by	
	One				1 year; improved with	
					heat therapy	
[48]	Case report	Male, 29 years	SSG 20 mg/kg		Partial clinical response	1987: LCL cured after
	Addis Ababa, 1993	Original lesion	30 days		EOT; cured at M8	SSG 30d;
	Recurrence of LCL	treated and cured			Parasitological failure	Recurrence (1991): no effect
	due to AIDS	in 1987			(no change)	effect of SSG
**Main focus pentamidine**						of SSG 30 days
[49]	Case series	6 males, 2 females	Pentamidine (no details)	2	DCL: both “responded	2 LCL & 1 lupoid CL Not
	DCL (2)/LCL(6)	Age range 12–30 years			well”	not parasitologically
	combined with			1	CL (lupoid): “responded	Confirmed
	leprosy;				well”	7/8 lepra cases treated
	Addis Abeba, Ethiopia			4	LCL: all “responded well”	(with dapsone)
	1965–67; 1974–76		Cycloguanil paomate	1	No info	Outcome: “responded
	No species identification					well”; relapse in 1 LCL
[50]	Case series,	Details lacking	Pentamidine (Lomidine) IM		General comments:	Despite dramatic
	20 complicated CL cases,	No sex predominance	+/- 1 cc/10 kg every		“Antimonials ineffective	improvement with,
	Ethiopia; 1965	Onset: age 4–12 years	2 d for 7 doses		(SSG 10 cc IM 17 days)”	recrudescence typically
	No species identification	except one 57 years			Pentamidine: “rapid	seen after pentamidine;
					diminution of lesions”	All parasitologicallly
						confirmed?
[51]	Case series, MCL (4)	3 males, one female	Pentamidine 150 mg IM	2	Pentamidine: “very	Antimonials used in 2
	Ethiopia, 1978	Age range 15–49 years	twice weekly for 8 weeks		good response”	Cases since no
	No species identification		MA (Glucantime)	2	MA: “no response”	pentamidine vailable
			no details			
[52]	Case series; LCL, DCL	104 pts; 40 females	Pentamidine mesylate		49 LCL and all 6 DCL	55 pts treated, 20 with
	ALERT, Ethiopia	64 males	(Lomidil) 2–4 mg/kg IM		were treated	severe toxicity;
	5/1981-4/1983	74% age 10–39	Alternate days		No efficacy data on LCL	15 withdrawn PM
	Species identification in part	98 LCL, 6 DCL	Duration not given		DCL: All clinically	(severe nausea, vomiting,
	20	DCL: mean duration			improved at discharge	hypoglycaemia)
		5 years			3 still smear(+)	3 diabetes mellitus; 1atient
**Various**						
[53]	Case series, Addis Abeba,	2 females, 3 males	Metronidazole 3x500 mg 4–8	5	No clinical or histological	Remission after
	Ethiopia, 1978; MCL –		Weeks		Changes	treatment with
	parasitologically					Pentamidine
	confirmed;					Isethionate
	No species identification					No serious side effects
[54]	Case series; LCL,	No details	Cycloguanil pamoate (CI-	30	20 pts were reviewed at	Not all cases
	Wollega province		501; Camolar)		FU: improved (2); no	parasitologically
	Ethiopia, 1968		2x2.5 ml IM (2x 350 mg =		change (8); ambiguous (3)	confirmed; Pts that
	No species identification		twice the dose for		ie both signs of	improved had not
			for malaria treatment)		worsening/improve	been parasitologically
			lower for children; repeated		ment; worsening (7)	confirmed
			at 6 weeks in 10 pts			
[55]	Case report, LCL case	51 year old non,	Ketoconazole dose of 200	1	EOT: lesion 50%	Minimal response with 4
	having lived in Tigray	lesion duration: 27	mg b.i.d. for 4 weeks		Reduction	weeks application of
	region, Ethiopia	Months			By 2.5 months after	Pentamidine
	Lesion onset 1982,				treatment: cure/lesion	dimethylsulfoxide gel
	Ketoconazole given 1984				healed	No relapse at 12M
	No species identification					
**Ethiopian/Eritrean migrants or travelers[**54**]**						
[56]	Case report	Two children (both	Ointment containing 15%	1	All parasitologically cured	Two developed allergic
	LCL in Ethiopian patients	female), one male	paromomycin sulphate and		at ten days	contact dermatitis
	migrated to Israel, 1987	One case first	12% methylbenzethonium		All clinically cured but (end of	
	Longstanding LCL (2	treated with keto	chloride in white soft		treatment extended in	
	years)	conazole 50mg/d	paraffin—applied twice		two (10–30 days)	
		for 4 weeks	daily for 10 days		since at day 10 no clinical	
		without success			improvement	
[57]	Case report, LCL	No details	Miltefosine—No details	1	Clinically cured	CL probably
						acquired in Egypt
[58]	Case report	38-year old,	Liposomal amphotericin B	1	Clinically improved by 3	On infliximab,
	LCL in Eritrean man	Immunosuppressed	60 mg/kg body weight in		weeks; clinically cured at	methotrexate,
	travelled to Germany	male patient	doses of 200 mg/day for 2		12 month follow-up visit	and prednisolone for
		6x5 cm facial lesion	2 days (2.5–3 mg/kg/d x			rheumatoid arthritis
			22); total dose 4.4 gram			

^a^If the study period is not given, the year of publication is represented; cases parasitologicallly confirmed unless specified otherwise; species confirmation done (*L aethiopica*) unless specified otherwise

ALERT: All Africa Leprosy Rehabilitation and Training Center; AHRI: Armauer Hansen Research Institute; DCL: diffuse cutaneous leishmaniasis; EOT: end of treatment; IM: intramuscular; INH: isoniazid; LCL: localised cutaneous leishmaniasis; LST: Leishmania skin test; MA: meglumine antimonite; PO: per os; 1M FU: one month follow up; SSG: sodium stibugluconate

Two prospective studies including outcomes with antimonials and/or cryotherapy were found. In a study from Tigray, clinical non-response was observed in 23 (15%) of the 154 treated (intralesional or systemic SSG). Failure rates were particularly high in MCL and DCL cases and all of the HIV cases relapsed. In a study in Southern Ethiopia with a main focus on cryotherapy, 20 cases not qualifying for cryotherapy were treated with systemic SSG but two (10%) did not respond clinically. This study mainly included LCL, and most of these were identified during active case finding, which could possibly have led to earlier CL diagnosis. This study is also the only formal evaluation of cryotherapy in Ethiopia, showing cure in 81% of cases. A large case series of cryotherapy from the same centre demonstrated cure rates of 60–70% [5]. In Kenya, three cases unresponsive to standard SSG treatment were successfully treated with a high dose (18–20 mg/kg twice daily for 30 days). *In vitro* data generally suggested a low susceptibility of *L aethiopica* to antimonials (Table 5).

**Table 5:** Drug susceptibility studies on *L. aethiopica*.

Ref	Assay; parasite strains	IC50 or ED50	Comments
[18]	Amastigote-macrophage *in vitro* model—CD1 mouse	IC50 (μg/ml ± SD)	MF: maximal efficacy against all three forms; followed by ampho against LCL; by PM against DCL/ML;
	derived PEMs	- Ampho: 0.16 ± 0.18	DCL/ML generally less sensitive
	Patient strains:	- MF: 5.88 ± 4.79	
	LCL: 8; MCL: 9; DCL: 7	- SSG: 10.23 ± 8.12	
		- Paromo: 13.63 ± 18.74	
[20]	Human leukemia monocyte cell line THP-1	ED50:	*L*. *aethiopica* less sensitive to SSG than *L donovani*
	MHOM/ET/72/L100 strain	- SSG: 25.3 μg SbV/ml;	
		- PM: 0.6 μM	
		- Paromo: 6.4 μM	
[19]	Promastigote assay; Amastigote-macrophage assay (peritoneal CD1 mouse macrophages)	Promastigote (ED50-μM)	Ampho most active (submicromolar concentration)
	MHOM/ET/84/KH strain	- MF: 1.16–2.76	Tested in parallel: L Major generally least susceptible, *L donovani* most susceptible
		- Edelfosine: 0.62–1.28	
		- Ampho: 0.11–0.24	
		Amastigote (ED50-μM)	
		- MF: 2.63–4.92	
		- Edelfosine: 1.15–2.92	
		- Ampho: 0.04–0.07	
[21]	THP-1 monocyte cell line	1) ED50: 4.0 μg/mL (pre-treatment); 21.9 μg/mL (at relapse);	ED50 for SSG high: 78.2 μg Sb/ml (pt 1); 55.0 μg (pt 2)
	Patient strains (DCL-Ethiopia); Two patients (1,2) treated with PM and subsequent relapse, improving on PM/SSG	2) ED50: 7.1 μg/mL (pre-treatment),: 21.3 μg/mL(at relapse)–	Sb/ml synergism with SSG in both patients
	Patient 3 treated with PM/SSG	3) ED50: 15.0 μg/mL (pre-treatment)	

ampho: amphotericin B deoxychelate; DCL: diffuse cutaneous leishmaniasis (CL); ED50: effective dose 50; IC50: inhibitory dose 50; LCL: localized CL; MCL mucocutaneous leishmaniasis; MF: miltefosine;; Paromo: paromomycin; PEM: Peritoneal exudate macrophages; PM: pentamidine; SSG: sodium stibogluconate; Sb: pentavalent antimonial.

A number of (often older) studies reported pentamidine—in contrast to antimonials—to be effective against CL (including MCL). However, this were typically case series, reporting on patients from routine clinical practice and without standardized outcome reporting. Pentamidine also appeared effective in the above mentioned clinical trial and in the complicated CL cases (CL relapse not responding to SSG including MCL) in the prospective study from Tigray [7]. We identified one case study of LCL treated with ketoconazole, with 50% lesion reduction at the end of treatment and cure 2.5 months later.

Several small studies reported on treatment in countries outside Ethiopia. Paromomycin ointment was highly effective in three Ethiopians treated in Israel. As to miltefosine, one case of successful treatment was reported in Germany. Of interest, miltefosine has been successfully used in more than 50 CL cases due to *L aethiopica* in Addis Ababa, but the findings have not been published (personal communication, Asrat Hailu). Liposomal amphotericin B was found effective in one immunosuppressed Eritrean patient treated in Germany.

Four studies included MCL patients. Metronidazole was ineffective in five cases of MCL. Antimonials were usually not found effective, but better outcomes were generally observed with pentamidine.

The available *in vitro* data relating to these drugs suggest a good susceptibility of *L aethiopica* to miltefosine, paromomycin, pentamidine and amphotericin B (Table 5). The most recent study by Utaile *et al* was conducted in Ethiopia using strains isolated from patients [18]. Parasite susceptibility was highest for AmBisome (in the sub-micromolar range), followed by miltefosine with an IC_50_ of 5.88 μg/ml. Efficacy of miltefosine against visceral leishmaniasis and other CL-causing species was exerted in a similar low micromolar range. Miltefosine had the highest maximal efficacy against CL, MCL and DL. Paromomycin had the highest IC_50_ but had the second highest maximal efficacy against MCL and CL strains. Two other studies used reference strains. In the study by Escobar *at al*, amphotericin B was again active at low concentrations; the ED_50_ for miltefosine was less than 5 μM [19]. In a third study, pentamidine had the lowest ED_50_ (0.6 μM), followed by paromomycin (ED_50_ 6.4 μM) [20]. In another study, paromomycin was evaluated against three strains, with ED_50_ of 4.0–15.0 μg/ml before treatment [21]. Antimonials were only active at high concentrations, but exerted synergism with PM which correlated with the clinical response.

### Clinical studies on DCL treatment

As to treatment with pentamidine, we found data on 62 patients in a total of ten studies (Table 6). The largest group of patients stems from pioneering work done by Bryceson and colleagues in the late sixties, reporting on 31 patients treated with varying regimens of pentamidine. Of 24 patients receiving pentamidine daily or every other day in the study by Bryceson, all improved during treatment although none was cured. However, frequent pentamidine administration was associated with substantial toxicity. With less frequent administration, safety was improved at the expense of efficacy. An additional four cases were reported in four other studies, all showing clinical improvement.

**Table 6:** Clinical studies specifically focused on diffuse cutaneous leishmaniasis due to *L. aethiopica*.

Ref	Study design; country; year; study population^a^	Patient characteristics	Drug/Intervention	N	Efficacy	Comments
[59]	Case series; DCL	Female, 28 years	Pentamidine	1	Clinical, histological	No info on toxicity
	Ethiopia; 1982	DCL for 2 years	dimethane sulphonate		and parasitological	Male pt had been
		Male, 35 years,	200 mg (Lomidine) daily		improvement by EOT	treated with
		DCL for 10 year	IM for two weeks		(no “cure”)	pentamidine before
						but had relapsed
[60]	Case series; DCL		Pentamidine dimethansulphonate 4 mg/kg	31	31 improved (7 cured)	Cure: clinic-
	Ethiopia; 1970		(Lomidine)			parasitological
	No species identification		- Daily or alternative days (4–23 doses); TD 1–3.4g	24	24 improved (0 cured)	resolution; Improved:
					13 relapse	clinical improvement;
			- Weekly (4–21 weeks); TD 0.7–2.4g	12	11 improved (1 cured)	Relapse common; Most
			- Every two weeks (2-10w)	9	6 improved (2 cured)	toxicity with PM &
			- Monthly (5–6 months)	5	3 relapses; 2 lost	amphotericin B; dose-
			Pentostam: 10–20 mg/kg 5–30 days, TD 3–17 gr	17	0 improved 0	limiting toxicity with
			Glucantime: 10–21 days; 12 mg/kg 10–21 days (TD	8	4 improved	frequently doses PM
			3–12.6 gr)			
			SSG + pentamidine	16	12 improved (4 cured)	Other drugs tested (no
			(for relapse after SSG)		6 relapsed	convincing effects):
			alternating 10 doses			high dose antimalarials;
			each (2–8 weeks)			astiban macrocylon,
			Amphotericin B 1 mg/kg on alternating days (29–48	4	4 improved 4 (1 cured)	Griseofulvine
			mg/kg TD over 3–5 months)			
[61,62]	Case report	40 year old female	PM isethionate 200 mg IM every		Marked reduction in reduclinimprovement,	Mention >100 CL cases
	DCL relapse after PM	DCL since 25 years	every 2 d for one month		parasite load; no DM	PM treated 1974–79
	Previously transient DM	Non-response to			Subsequent relapse	PM isethionate 200 mg IM
	with Lomisil (pentamidine	MA, metronidazole			treated with Lomisil:	daily 15 days: no DM
	mesylate)	dapsone,			developed DM	Lomisil period 1979–80
	AHRI, Ethiopia; 1978	Chloroquine				DM in 2/30 in 2 year
		Clofazimine				
[21]	Case series, DCL	Male (2)	Paromo 14 mg/kg 60 days	2	EOT complete clinico-	Relapse after 1 and 3
	Addis Ababa,		(2 cases of DCL)		parasitological resolution	months; subsequently
	Ethiopia, 1990–1992		(reduced to 12 mg/kg			Treated with paramo +
	No species identification		3x/weeks in one pt due to			SSG
			renal dysfunction			
		Male (2); female (1)	Paromo 14 mg/kg + SSG	3	EOT complete clinico-	Relapse free at 17, 2 and
			10 mg/kg 2 months-		parasitological resolution	21 months; Audiograms
			beyond parasitological cure			normal
			(6–9 months; 2 patients			PM dose reduction in
			relapsing after paromo (see			one patient for renal
			above) + 1 new patient			Dysfunction
[63]	Case series, DCL	Three (no details)	Chlorpromazine 2%	3	All became smear neg	No long term outcome
	Addis Ababa,		ointment for one month		Inflammation dis-	All three confirmed
	Ethiopia; 1983				appeared (3); regression	parasitologically
	No species identification				of affected area (1)	
[53]	Case series, DCL	Two (no details)	Metronidazole—no details	2	Initial clinical	No complete remission
	Addis Ababa; 1978		Given		improvement;	
	No species identification				histologically confirmed: 1	
					1	
[64]	Case series, DCL	Atypical DCL (n = 2);	MA 28 days IM	2	Clinical improvement	One case received
	Mekele; 2013	alike borderline-			EOT	a second treatment
	No species identification	tuberculoid leprosy				course for consolidation
		Males (18, 20 years)				

^a^If the study period is not given, the year of publication is represented; cases parasitologicallly confirmed unless specified otherwise; species confirmation done (*L aethiopica*) unless specified otherwise

ALERT: All Africa Leprosy Rehabilitation and Training Center; AHRI: Armauer Hansen Research Institute; DCL: diffuse cutaneous leishmaniasis; EOT: end of treatment; IM: intramuscular; INH: isoniazid; LCL: localised cutaneous leishmaniasis; LST: Leishmania skin test; MA: meglumine antimonite; PO: per os; 1M FU: one month follow up; SSG: sodium stibugluconate

Outcomes with systemic antimonials were reported in 38 cases, of which only nine demonstrated clinical response. A prolonged course of paromomycin was successful in treating two cases of DCL, although both relapsed [21]. Three patients (including these two relapse cases) have been successfully treated with an extended course of paromomycin combined with SSG. Good susceptibility to paromomycin, poor susceptibility to antimonials but synergism between SSG and paromomycin could be demonstrated *in vitro* [21]. In the study by Bryceson, sixteen individuals were treated with antimonials and pentamidine in combination. Twelve improved during treatment, seven were declared cured. In the same study, all four cases treated with conventional amphotericin B treatment improved, but toxicity was substantial.

Metronidazole was tried in two patients, who displayed clinical improvement. As mentioned above, itraconazole was generally not found to be effective. Chlorpromazine ointment displayed some effect in a small case series, reported over 30 years ago.

## Discussion

The evidence base for treatment of CL due to *L aethiopica* remains extremely poor. Most studies were conducted decades ago, including often involving a few patients, lacking appropriate control groups and usually not employing a well-defined and rigid methodology. Outcome measures varied widely across the different reports. In drug susceptibility studies, pentamidine, paromomycin, amphotericin and miltefosine looked most promising. Antimonials were only effective *in vitro* at relatively high doses. Some studies supported the efficacy of antimonials against LCL, others reported on the poor efficacy. Both the national and WHO recommend topical or systemic administration of antimonials for LCL treatment in Ethiopia (Table 7) and, despite the lack of a good evidence base, antimonials remain the most widely available anti-leishmanial drug in Ethiopia. Given the common and sometimes severe toxicity of antimonials, better defining its efficacy against CL in clinical trials is urgently needed.

**Table 7:** Ethiopian and WHO guidelines for treatment of cutaneous leishmaniasis in Ethiopia.

	National guidelines	WHO guidelines
Local Therapy	- Intra-lesion SSG weekly for four to six weeks	Several options but indicating limited evidence base
	- Cryotherapy	- Cryotherapy or intra-lesional
	- Curetage^a^	SSG (alone or combined)
	- Heat therapy^a^	- Thermotherapy
	- Topical ointment^a^	- Topical ointment
Systemic therapy	- Paromomycin 14–15mg (sulphate)/ kg for 20–30 days	- 20 mg of Sb5+/kg/d IM or IV for 28 days
	- 20mg Sb5+/kg/day IM or IV for 4–8 weeks	
	- Miltefosine^a^	
	- Liposomal amphotericin B^a^	
Diffuse CL	- Paromomycin 15mg/kg + SSG 10mg/kg	- 20 mg of Sb5+/kg/d IM or IV for 28 days + Paromomycin 15 mg/kg/day IM for 60 days or longer (C)
		- Pentamidine isethionate 3–4 mg/kg/d IV once or twice weekly (up to 4 months) (C)

Level of Evidence (C): Opinions of respected authorities, based on clinical experience, descriptive studies or reports of expert committees

^a^ guideline stipulates these have not yet been used for CL in Ethiopia

CL: cutaneous leishmaniasis; IM: intramuscular; IV: intravenous; SSG: sodium stibugluconate

Sb5+: pentavalent antimonial

The national guidelines recommend intramuscular paromomycin as the preferential LCL first line treatment. This is backed-up by *in vitro* data, although clinical evidence is very scarce with only a small case series on DCL and no reports on LCL caused by *L aethiopica*. Its good safety profile and availability in Ethiopia argue for its evaluation in clinical trials. At a dose of 12–18 mg/kg/day for 14 days, cure rates of over 90% were obtained in Brazil [22], but only 50–60% in Colombia and in Belize [23,24].

Clinical efficacy of miltefosine has varied globally according to the etiological species and geographical area. In drug susceptibility testing, miltefosine looked promising against *L aethiopia*, warranting clinical studies. Miltefosine is now increasingly available in East-Africa for VL treatment, creating opportunities for CL as well. Few studies are available on the use of miltefosine against other species causing Old World CL. In one trial from Iran, miltefosine was found as effective as intralesional antimonials against CL caused by *L major* [25]. As to New World LCL, the efficacy of miltefosine varied across regions and/or species [26].

Although pentamidine appeared effective in antimonial-resistant complicated CL, given its safety profile, it would probably only be considered as second line treatment for LCL. Given their ready availability in resource-limited settings (often via HIV programs), the efficacy of drugs such as ketoconazole and fluconazole merit further exploration.

Data on the efficacy of topical and physical therapies are very limited. Only cryotherapy and intralesional antimonials have to some extent been evaluated against CL in Ethiopia. Other topical (e.g., paromomycin) and physical therapies might be worth exploring, particular if easy to administer and if implementable in remote settings with limited well-trained health care staff. Recent findings with thermotherapy from Peru are modestly encouraging [27]. Topical application of amphotericin B is currently in early clinical evaluation by the Drugs for Neglected Diseases initiative (DND*i*) against *L braziliensis* and *L tropica* (www.dndi.org). If promising, this should be expanded to *L aethiopica*.

Treatment of DCL has been notoriously difficult. While there have been only a few studies on pentamidine, available data seem to indicate a relatively good efficacy, although relapse was commonly observed after pentamidine discontinuation. While toxicity has been a critical concern issue with daily administration (for instance as treatment for human African trypanosomiasis), less frequent administration (e.g., every other day) appeared to be well tolerated while still effective. The WHO recommendations also include pentamidine for DCL treatment. Nevertheless, cumulative toxicity remains a concern, especially as to the risk of diabetes [28,29]. Eight cases of diabetes have been reported from Ethiopia, presumably related to pentamidine use, also in cases that received less frequent administration. However, it is important to note that in the earlier studies pentamidine mesylate (Lomidine) was used, labelled according to the base-moiety (120 mg base per ampoule) with a recommended dose of 4 mg base/kg body weight. Currently, pentamidine isethionate (Pentacarinat or Pentam) is used, labelled according to the amount of salt in the preparation (300 mg salt per ampoule) with a recommended dose of 4 mg of salt/kg. In practical terms, this means that earlier studies employed a higher dose (7 mg of salt/kg) than currently being advised by the company (4 mg of salt/kg) [30]. While possibly leading to improved tolerance, it is unclear to what extent efficacy would be compromised. Nevertheless, the potential risk of diabetes and other adverse effects requires close clinical and laboratory monitoring, restricting its’ use to better established health facilities.

Both the WHO and national guidelines include the combination of paromomycin and antimonials for DCL treatment, albeit with different dosing of the antimonials and acknowledging the limited evidence base. This combination now constitutes the first line VL treatment in VL endemic east African countries, which would facilitate its implementation if found effective. Nevertheless, a daily parenteral treatment regimen of at least two months remains cumbersome and can only be seen as a short-term solution. Paromomycin and miltefosine in combination would also be worth evaluating. Miltefosine was found effective against DCL in a small study conducted in Venezuela, with most cases caused by *Leishmania amazonensis*, although all but one subsequently relapsed [31]. This combination (given for ten days) has been found safe and effective against visceral leishmaniasis in India [32].

Even if more potent drugs for DCL would be identified, most cases are likely to relapse after treatment discontinuation, unless the underlying immunosuppression can be altered. This provides the rationale for complementary approaches such as adjuvant immunotherapy. Effective treatment of DCL will most likely require adjuvant strategies such as immunotherapy to consolidate the treatment response. Therapeutic vaccination with first generation vaccines—has been found effective against DCL and ML in the New World [33,34], but has not yet been explored in Ethiopia. Moreover, second generation vaccines have been developed [35], which looked promising in phase I studies against New World CL and ML [36,37]. DNDi (www.dndi.org) is currently exploring the use of CpG-DNA [38–42] for CL due to *L braziliensis* and *L tropica*. An interesting approach could combine chemotherapy with therapeutic vaccination with a second generation vaccine and/or CpG-DNA against the different forms of CL.

Trials should probably initially focus on LCL and possibly MCL, since this is the predominant clinical presentation. However, efforts should also be made to provide treatment to more complicated manifestations within clinical trials such as DCL (e.g., via a compassionate use protocol in a well-documented case series). Given the lack of quality data on any of the available treatments, several potentially interesting interventions should be evaluated in future clinical trials, with the most promising ones taken further in more extensive evaluations. Adaptive clinical trial designs have been increasingly used in NTDs, since these have the potential to more quickly and efficiently weed out ineffective regimens across the different intervention arms [43,44]. For non-complicated LCL, various topical or physical therapies should be evaluated.

As to systemic treatment for complicated CL, interventions to be prioritized for evaluation include paromomycin, miltefosine and antimonials (possibly combined with paromomycin). While the higher cost of liposomal amphotericin B is an obvious disadvantage, price reductions (e.g. via generics) remain possible and the drug is increasingly available in VL endemic countries [45].

In conclusion, the evidence-base for treatment of CL due to *L aethiopica* is extremely limited, warranting prospective clinical studies. While antimonials might for the time being remain the cornerstone of CL treatment, not in the least because of their availability and clinical experience within Ethiopia, their efficacy and safety in CL should be better defined. Most importantly, alternative first line treatments should be explored, preferably topically or to be taken orally. As to DCL, the options appear limited. Pentamidine appears most promising, but toxicity is an issue. High quality trials on CL due to L aethiopica are urgently needed. A good scenario would be several options in parallel using adaptive designs/group sequential methods to discontinue the arms with ineffective drugs.

## Supporting Information

S1 Checklist PRISMA Checklist. (DOC)
